# The effect of consuming voluntarily fortified food and beverages on usual nutrient intakes in the Canadian population

**DOI:** 10.29219/fnr.v65.5256

**Published:** 2021-06-04

**Authors:** Valerie Tarasuk, Didier Brassard

**Affiliations:** 1 Department of Nutritional Sciences, University of Toronto, Toronto, Canada; 2 Centre Nutrition, santé et société (NUTRISS), Institut sur la nutrition et les aliments fonctionnels (INAF), Université Laval, Québec, Canada

**Keywords:** voluntary fortification, dietary assessment, usual intakes, National Cancer Institute Method, Canada

## Abstract

**Background:**

In Canada, regulatory changes have expanded marketing opportunities for voluntarily fortified products (VFPs), with micronutrient additions permitted at levels well in excess of human requirements.

**Objective:**

To examine how the consumption of VFPs relates to usual nutrient intakes in the Canadian population.

**Design:**

The 2015 Canadian Community Health Survey comprises single 24-h dietary intake recalls on a population-representative sample of 20,487 individuals aged 1 year and older, with second recalls on a subset of 7,608. The intake data included 15 food codes denoting VFP (e.g. energy drinks, fortified beverages, cereals, and bars). We assessed VFP consumption and estimated usual intake distributions for riboflavin, niacin, zinc, and vitamins A, B6, B12, and C for VFP consumers and non-consumers 14–50 years old (*n* = 8,442) using the National Cancer Institute method. We applied the ‘shrink and add’ method to estimate usual intakes among supplement users and assessed apparent benefits and risks by comparing usual intake distributions to EARs and ULs.

**Results:**

Only 2.4% of the population reported any consumption of VFP on the first 24-h recall. VFP consumers were overrepresented in the upper quartile of population intake distributions for niacin, riboflavin, vitamin B6, vitamin B12, and zinc. The median usual intakes of VFP consumers were 24–111% higher than the median usual intakes of non-consumers, and VFP consumers had significantly lower prevalence of inadequacy for riboflavin and vitamins A, B6, B12, and C. Irrespective of VFP consumption, usual intake distributions reached the ULs for vitamin A and zinc with the addition of supplement intakes.

**Discussion:**

Given the limited differentiation of VFP in this survey, we have likely underestimated nutrient exposure levels.

**Conclusions:**

VFP consumption was associated with elevated usual nutrient intakes, but we found limited evidence that it protected consumers from nutrient inadequacies or propelled intakes above tolerable upper levels.

## Popular scientific summary

- In Canada, voluntarily fortification denotes the addition of vitamins and minerals to foods and beverages for marketing purposes, not as a public health intervention;
- People consuming voluntarily fortified products had 24–111% higher vitamin and mineral intakes than non-consumers, but how much this benefited them is unclear;
- We found little indication that the voluntarily fortified product consumption afforded valuable protection from risks of inadequate nutrient intakes, or that it increased the probability of intakes above tolerable upper intake levels.

Regulatory changes, in many cases prompted by trade agreements, have led to an expansion of voluntary fortification in many jurisdictions, including the European Union and Canada (1–3). This practice, also sometimes termed ‘liberal’ or ‘discretionary’ fortification, refers to the addition of vitamins and minerals at the discretion of food manufacturers. In countries where fortification for public health reasons (4) is tightly regulated, voluntary fortification denotes the addition of nutrients by manufacturers for marketing purposes. It is not part of a planned public health intervention.

In Canada, the voluntary addition of select nutrients to breakfast cereals has long been permitted, but regulatory changes introduced in 2003 (5) created opportunities for the sale of a broader array of voluntarily fortified products (VFPs), including energy drinks and other nutritionally enhanced beverages and bars. An updated regulatory framework now governs nutrient additions to caffeinated energy drinks (6) and other ‘supplemented’ foods and beverages (7, 8). Similar to regulatory approaches in other jurisdictions, nutrient additions to approved products are only limited by safety concerns. This is a marked departure from past fortification practices in Canada. Canada’s mandatory fortification programs require nutrient additions to specific foods to address defined public health problems (e.g. the mandatory addition of folic acid to enriched flour and grain products as a means to reduce the incidence of neural tube defects), for restoration purposes (e.g. thiamine, niacin, riboflavin, and iron additions to white flour), or to achieve nutrient equivalencies in ‘substitute’ foods (9). In contrast, voluntary fortification regulations allow a much wider array of nutrients to be added, with nutrient additions permitted at levels well in excess of naturally occurring nutrient concentrations in whole foods and irrespective of any evidence of nutrient needs in the population (6, 8). A recent examination of voluntarily fortified beverages marketed under these regulations revealed that nutrient concentrations were many times higher than current requirement estimates, but generally far below permitted maximums (10). As manufacturers seize the opportunity to market highly fortified products (10, 11) and their sales grow (12, 13), it is important to assess the impact of these products on nutrient exposures in the population.

There have been several studies examining the impact of voluntary fortification on nutrient intakes in the population, but the interpretation of this research is complicated by interjurisdictional differences in what constitutes voluntary fortification. Research from the United States and Ireland has highlighted the contributions of voluntarily fortified foods to nutrient adequacy (14–17), but the fortification practices assessed in these studies include nutrient additions that fall under mandatory regulations in many other jurisdiction (e.g. the fortification of flour and grain products with folic acid). One study that attempted to isolate the effects of voluntary fortification in the United States that was unrelated to public health needs indicated the potential for excessive nutrient exposures from this practice (18).

Drawing on 24-h dietary intake recall data from the 2015 Canadian Community Health Survey-Nutrition (CCHS-Nutrition 2015), this study was undertaken to 1) describe current consumption behaviors with respect to voluntarily fortified foods and beverages, 2) assess the relationships between voluntarily fortified food and beverage consumption and the consumption of micronutrient supplements, and 3) determine how the consumption of voluntarily fortified foods and beverages relates to usual vitamin and mineral intakes.

## Methods

The 2015 CCHS-Nutrition was a dietary intake survey of a population-representative sample of 20,487 individuals, aged 1 year and over, excluding residents of the territories, members of the armed forces, and those living on First Nations reserves, in remote communities, and in institutions (19). All survey participants completed one computer-assisted, interviewer-administered, 24-h dietary intake recall. The recall interview followed the USDA’s Automated Multi-pass Method, which has been extensively validated (20). A second 24-h recall was administered by telephone to a random subsample of 7,608 participants. A food booklet was provided to respondents to facilitate portion size estimation of foods and beverages in plates, bowls, glasses, and mugs. We identified voluntarily fortified foods and beverages potentially available for consumption in Canada through a review of current policies (7, 8) and publicly available lists of products currently authorized for sale (21). We then searched the food codes assigned to the 24-h dietary intake recall data in CCHS-Nutrition 2015 to identify matches to the products permitted for sale. Highly fortified breakfast cereals were identified through the ‘supplemented bars, shakes and meal replacements’ category using the Bureau of Nutritional Sciences food group codes (22). This search yielded discrete codes for four energy drinks, four meal replacement bars, one meal replacement cereal, and six nutrient-enhanced waters (Supplementary Table 1). Consumption behaviors for each product type were summarized for each dietary reference intakes (DRIs) age-sex grouping, considering the proportion of respondents reporting any consumption on the first 24-h recall. Because 74.8% (95% CI 67.0–82.7) of consumers were 14–50 years of age, we restricted subsequent analyses to participants in this age range (*n* = 8,442). Focusing on consumers in this narrower age range yielded a more homogeneous sample, while still retaining sufficient cell sizes to support our analyses.

**Table 1:** Sociodemographic and behavioral characteristics of the analytic sample (*n* = 8,442)

	Non-consumers, *n* = 8,135	Consumers, *n* = 307	*P* ^a^
**Sex**			0.0090
Male	49.6%	64.8%	
Female	50.4%	35.2%	
**Age group**			0.0041
14–18 years	11.2%	14.4%	
19–30 years	26.3%	40.7%	
31–50 years	62.5%	44.9%	
**Education**			0.9417
No diploma	13.8%	14.4%	
High school completion	23.9%	26.6%	
Trade school, some college	29.0%	26.8%	
University	33.3%	32.2%	
**Household income, before taxes**			0.0473
<20,000 $CAD	6.2%	3.3%	
20,000 to <40,000 $CAD	13.2%	6.3%	
40,000 to <60,000 $CAD	14.2%	14.9%	
60,000 to <80,000 $CAD	13.4%	13.9%	
80,000 to <100,000 $CAD	13.3%	8.9%	
100,000 $CAD and higher	39.7%	52.8%	
**Current smoking status**			0.2622
Not smoking	82.2%	74.0%	
Occasionally smoking	5.5%	11.5%	
Daily smoking	12.3%	14.5%	
**Physical activity**			0.0055
Did not meet guideline	55.6%	36.7%	
Met the physical activity guideline (150 min/week)	44.4%	63.3%	
**Micronutrient supplement intake^b^**			0.0816
Did not consume supplement	61.7%	70.3%	
Consumed a supplement of interest	38.3%	29.7%	
**Weight status**			0.4365
Underweight, BMI < 18.5 kg/m^2^	4.7%	2.3%	
Normal, 18.5 ≤ BMI ≤ 24.9 kg/m^2^	42.6%	39.7%	
Overweight, 25 ≤ BMI ≤ 29.9 kg/m^2^	29.6%%	36.4%	
Obese, ≥ 30 kg/m^2^	23.0%	21.6%	
**Plausibility of reported energy intake^c^**			0.3590
Underreported	32.5%	27.5%	
Plausible or over-reported	67.5%	72.5%	

a P-values are Rao-Scott modified chi-square tests, showing compatibility of frequencies with the (null) hypothesis of no difference between consumers and non-consumers.

b Consumption of a supplement providing vitamins A, C, B6, and B12, riboflavin, niacin, or zinc.

c Plausibility of reporting was assessed by comparing the ratio of energy intake: total energy expenditure to survey-specific cut-offs (24).

We compared the sociodemographic, behavioral, and anthropometric characteristics of consumers and non-consumers, considering age, sex, education, income, smoking status, physical activity level, supplement use, weight status, and energy misreporting, and applying Rao-Scott modified chi-square tests to compare distributions between groups. Weight status was determined using standard thresholds for body mass index (19). The prevalence of energy misreporting was calculated for VFP consumers and non-consumers by first expressing each participant’s energy intake from the first 24-h recall as a ratio of their total energy expenditure (TEE) (23) and then comparing this ratio to standard cut-points denoting under-, plausible, and over-reporting (24). Standard sex- and age-specific equations for TEE included variables for age, height, weight, and physical activity level (23). Given the lack of an objective physical activity assessment in CCHS-Nutrition 2015, a sedentary level of physical activity was assumed for all respondents. This assumption may cause an overestimation of over-reporting but permits a more sensitive assessment of underreporting. The cut points applied to assess reporting status were developed specifically for use with CCHS-Nutrition 2015 and follow standard assumptions (25–27), taking into account within-person variation in energy intake and expenditure and error in the predictive equations for TEE (24).

Our analyses of micronutrient exposure focused on riboflavin, niacin, zinc, and vitamins A, B6, B12, and C. These seven nutrients were selected because they are found in both energy drinks and supplemented foods (10), with additions permitted in substantial amounts for at least some of these products (6, 8), and the nutrients are all included in Canada’s food composition database, the Canadian Nutrient File 2015 (28). The contribution of VFP to total energy and micronutrient intake on the first 24-h recall was described, considering absolute amounts and percent contribution to total intake with these values calculated for each individual 24-h recall. We also examined the distribution of absolute micronutrient intakes from the products on the recall day, recognizing the potential adverse health effects from acute exposure to some of the added nutrients (29).

To explore the effect of VFP consumption on relative micronutrient intake levels, we calculated the proportion of consumers above the highest quartile of intake for their respective age/sex group for each micronutrient, using data from the first 24-h recalls. Odds of being above the highest quartile according to VFP consumption were calculated using logistic regression and the cumulative logit function, adjusting for total energy intake, age, and sex. Since a large proportion of VFP consumers had intakes above the highest quartile, odds ratios were transformed to approximate prevalence ratios using the method described by Zhang and Yu (30).

To garner some insight into how the reported consumption of VFP on a single 24-h recall related to habitual intake, we examined the relationship between the reported VFP consumption on first and second 24-h recalls. Considering only survey participants aged 14–50 years who completed two recalls, we applied the Rao-Scott modified chi-square test to compare proportions reporting VFP on the second recall compared with the first recall. We observed a much greater likelihood of VFP consumption on the second recalls of those who reported consumption on the first recall. Rather than treating these products as episodically consumed foods in the subsequent analysis of usual intakes (18) (an approach that presumes some probability of consumption across all members of the population), we elected to consider VFP consumers, identified from their reported consumption on the first 24-h recall, as a discrete subset of the population (*n* = 307).

To further explore the effect of VFP intake on total nutrient loads, we compared the first and second 24-h recalls of all 14–50-year-old participants with two 24-h recalls by the presence of VFP on these recalls. We hypothesized that among individuals who consumed VFP on only one of the two recall days, VFP consumption would be associated with significantly higher nutrient intakes. Days 1 and 2 intakes were compared within four discrete subgroups: participants who consumed VFP on both recalls, those who did not consume VFP on either day, those who consumed VFP on the first but not the second recall, and those who consumed VFP on the second but not the first recall. Percentage differences in nutrient intakes between days 1 and 2 were calculated as 100*ln (intake on day 2) − 100*ln (intake on day 1), with nutrient intakes expressed as natural logarithms to increase the comparability of the two measurements, since dietary intakes on a given day are heavily skewed (31). Within each of the four subgroups, least-square mean percentage differences and 95% confidence intervals were derived from linear regression models including covariates for age, sex, and energy intake. Energy was included in these models to adjust for differences in total intake between the 2 days of recall data, recognizing the trend toward lower energy intakes on the second dietary intake recall that was administered by telephone. Mean differences were then tested against the null hypothesis that the difference in nutrient intakes between the 2 days was 0.

We estimated the distributions of usual vitamin and mineral intakes for consumers and non-consumers using the National Cancer Institute (NCI) method (MIXTRAN and DISTRIB). Because of the low prevalence of reported consumption of VFP, we pooled data for males and females 14–18, 19–30, and 31–50 years of age to increase the stability of the estimated distributions of usual nutrient intakes for consumers. Each model included covariates for age and sex as well as binary variables to denote whether the dietary data were from the first or second recall and whether they were collected on a weekday or weekend.

The prevalence of vitamin and mineral supplement use among consumers and non-consumers of VFP was compared, first considering the supplement use as a simple binary variable based on reported consumption of a dietary supplement that contained at least one of the seven micronutrients of interest in the last 30 days. The usual intake distributions of consumers and non-consumers were then reestimated to include nutrients from supplements by adding average usual daily nutrient intakes from supplements over the last 30 days to the predicted usual intakes from the diet [i.e. the ‘shrink then add’ approach (32)].

Finally, to assess the implications of current VFP consumption patterns for nutrient adequacy and excess, the distributions of usual nutrient intake constructed above were compared to current age- and sex-specific estimated average requirements (EARs) and tolerable upper intake levels (ULs), if applicable (33–35), applying the EAR cut point method to estimate the prevalence of nutrient inadequacies. In our assessment of nutrient adequacy, we did not account for the added requirements of women who were pregnant or lactating; they comprised a very small fraction of our sample (i.e. 2.8% of all 14–50-year-old respondents). Because we could not differentiate nicotinamide from other forms of niacin, risk of excessive intakes was not assessed for this nutrient.

Using weights provided by Statistics Canada, all analyses were weighted with 500 bootstrap replications using the bootstrap weights to calculate variance and sampling weights to be representative of the Canadian population. Determinations of statistical significance were based on *P*-values < 0.05 and comparisons of 95% confidence intervals.

## Results

**Fig. 1:**
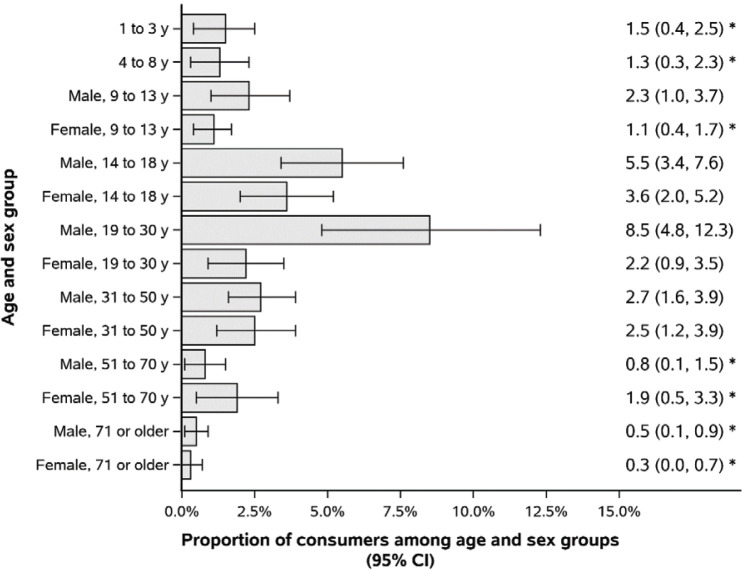
Proportion of population reporting consumption of any voluntarily fortified product on the first 24-h recall within each age and sex group. All data are weighted to be representative of the Canadian population. *Estimate with high uncertainty as per Statistics Canada’s standard (coefficient of variation > 33.3%).

On a single 24-h recall, 2.4% (95% CI: 1.9–2.9) of the population reported some consumption of VFP, with the prevalence ranging from 0.3% (95% CI: 0.0–0.7) among females 71 years and older to 8.5% (95% CI: 4.8–12.3) among males 19–30 years old (Fig. 1). Of the products reported in the entire population, 28.9% were meal replacement bars, 28.7% were meal replacement cereals, 23.5% were energy drinks, and 19.0% were nutrient-enhanced waters. When only the intakes of individuals 14–50 years of age were considered, the distribution of VFP products consumed shifted slightly: 30.7% were energy drinks, 29.0% were meal replacement bars, 23.1% were meal replacement cereals, and 17.3% were nutrient-enhanced waters.

The characteristics of VFP consumers and non-consumers 14–50 years of age are presented in Table 1. The groups differed significantly with respect to sex, age, income, and physical activity. Of note, 35.2% of consumers were female, compared to 50.4% of non-consumers, and the average age of consumers was 30.0 years, compared to 33.9 years for non-consumers. Consumers also tended to have higher household incomes and to be more physically active. Both the prevalence of supplement use and the estimated prevalence of energy underreporting were higher among non-consumers than VFP consumers, but these differences were not statistically significant.

Table 2 provides a summary of the distribution of nutrient intakes from VFP among 14–50-year-old consumers in absolute terms and as a proportion of total nutrient intake on a single 24-h recall. There was marked variation in the nutrient amounts that individuals obtained from VFP, reflecting between-person differences in product selection and quantities consumed. The 75th centile of intakes from VFP exceeded the EARs for all of the B vitamins examined here, but only the 95th centile of intakes for vitamin C exceeded the EARs, and even this point on the distribution fell below the EARs for vitamin A and zinc. Although VFP comprised, on average, 8.3% of consumers’ total energy intake, their mean contribution to total micronutrient intakes ranged from 11.7% for vitamin A to 50.6% for vitamin B6 (Table 2).

**Table 2:** Distribution of nutrient intakes from voluntarily fortified products and percent contribution of VFPs to total nutrient intakes on the first 24-h recall, among consumers 14–50 years of age

Nutrient	EAR^a^	Mean (SD)	5th centile	25th centile	50th centile	75th centile	95th centile	Mean contribution to total intake, %	Mean total intake (95% CI)
Energy, kcal		181 (179)	10	83	137	243	514	8.3	2,198 (1,971, 2,426)
Vitamin A, µg retinol activity equivalents	485–625	89.4 (131)	0	0	50.9	152	320	11.7	765 (648, 881)
Vitamin C, mg	56–75	59.9 (95.3)	0	0	14.4	46.6	256	40.2	149 (121, 177)
Niacin, mg	11–12	14.5 (22.3)	0.4	5.2	7	18.6	36.7	25.0	58 (52, 65)
Riboflavin, mg	0.9–1.1	0.9 (2.0)	0	0	0.4	1.1	3.4	30.8	3.0 (2.6, 3.4)
Vitamin B6, mg	1.1	1.8 (3.0)	0.1	0.3	0.8	2.6	5.6	50.6	3.6 (3.1, 4.1)
Vitamin B12, µg	2.0	2.3 (6.0)	0	0	0.7	3.0	8.6	37.5	6.2 (5.3, 7.2)
Zinc, mg	6.8–9.5	1.9 (2.9)	0	0	0.7	3.1	6.4	14.5	13.1 (11.3, 14.9)

All data are weighted to be nationally representative. A total of 307 respondents (representing 2.4% of the Canadian population) reported the consumption of voluntarily fortified products. SD, finite population standard deviation.

a Estimated average requirements; range reflects age/sex differences in requirement estimates among 14–50 year olds.

**Fig. 2:**
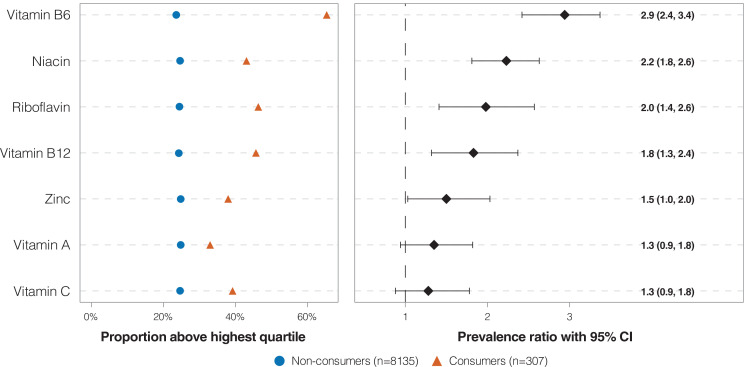
Proportions and prevalence ratios of Canadians, 14–50 years of age, with intakes above the highest quartile of nutrient intake on the 1st 24-h recall, by the consumption of voluntarily fortified products. All data are weighted to be representative of the Canadian population. Age- and sex-specific quartiles of nutrient intakes were used to categorize respondents, regardless of consumption of voluntarily fortified products. Logistic regression models were adjusted for age, sex, and total energy intakes. Cumulative-logit models were used, and the probabilities modeled were summed over the responses having the lower ordered groups (quartiles 1, 2, and 3).

The prevalence of VFP consumers above the upper quartile of nutrient intakes for the population based on the first 24-h recall ranged from 32.9% (95% CI: 22.5–43.4) for vitamin A to 65.3% (95% CI: 54.6–75.9) for vitamin B6 (Fig. 2). After adjustment for age, sex, and energy intake, significantly elevated prevalence ratios were observed for vitamin B6, niacin, riboflavin, vitamin B12, and zinc, but not for vitamins A and C (Fig. 2).

Among the 2,550 participants who completed two 24-h recalls, 25.1% (95% CI: 11.1 to 39.1) of those who reported the consumption of a VFP on the first recall also reported such consumption on the second recall. Only 1.7% (95% CI: 0.9–2.4) of participants who reported no VFP consumption on the first recall reported such consumption on the second recall. In other words, people who consumed VFP on the first recall had a prevalence of VFP consumption on the second recall that was 14.8 times higher than the prevalence of among those who reported no VFP on the first recall (*P* = 0.0062).

Among the participants who completed two 24-h recalls but reported VFP consumption on only one recall day, intakes of several nutrients were significantly higher on days when VFPs were consumed (Table 3). Specifically, individuals who consumed VFP on day 1 but not day 2 had significantly lower intakes of every nutrient examined except vitamin C on day 2. Individuals who consumed VFP only on day 2 had significantly higher intakes of vitamin B6 and niacin on day 2 compared to day 1. The nutrient intakes of individuals who consumed VFP on both days did not differ significantly between days, and among individuals who did not consume VFP on either day, the only significant difference detected was a 5% decrease in riboflavin intakes on day 2.

**Table 3:** Nutrient intake difference between the first and the second 24-h recalls, by voluntarily fortified product (VFP) consumption, in 14–50-year-old Canadians who completed two recalls

	VFP consumption on both days, % difference^a^ (95% CI)	*P* ^b^	VFP consumption on day 1 only, % difference^a^ (95% CI)	*P* ^b^	No VFP consumption on either day, % difference^a^ (95% CI)	*P* ^b^	VFP consumption on day 2 only, % difference^a^ (95% CI)	*P*-value^b^
Vitamin A	−35 (–90, 20)	0.2135	−58 (−87, −28)	0.0002	−4 (−14, 6)	0.3988	−4 (−42,34)	0.8380
Vitamin C	10 (–49, 69)	0.7445	−43 (−100, 14)	0.1424	−5 (−18, 8)	0.4234	19 (−27, 65)	0.4195
Niacin	–11 (–52, 29)	0.5891	−48 (−75, −20)	0.0007	0 (−5, 5)	0.9552	20 (0, 39)	0.0467
Riboflavin	–9 (–47, 29)	0.6350	−56 (−86, −26)	0.0003	−5 (−10, −1)	0.0250	9 (−18, 36)	0.5175
Vitamin B6	–37 (–82, 9)	0.1138	−94 (−126, −62)	<0.0001	1 (−5, 6)	0.7916	48 (22, 73)	0.0003
Vitamin B12	0 (–47, 46)	0.9879	−62 (−102, −21)	0.0028	2 (−7, 10)	0.7145	10 (−20, 40)	0.4944
Zinc	–13 (–45, 19)	0.4165	−21 (−41, −1)	0.0400	−3 (−9, 3)	0.2873	−11 (−34, 13)	0.3775

a Values are least-squares mean percentage differences (95% CI) in intakes on the second 24-h recall minus intake on the first 24 our recall, derived from a linear regression model adjusting for age, sex, and 24-h total energy intake. A negative difference indicates that intakes on day 1 were greater than intakes on day 2, and vice-versa. Percentage differences were calculated as 100*ln (intake on day 2) - 100*ln (intake on day 1).

b P-values are partial hypothesis tests indicating compatibility of the observed day 2 versus day 1 difference with the (null) hypothesis that the day 2 versus day 1 difference is zero.

**Fig. 3:**
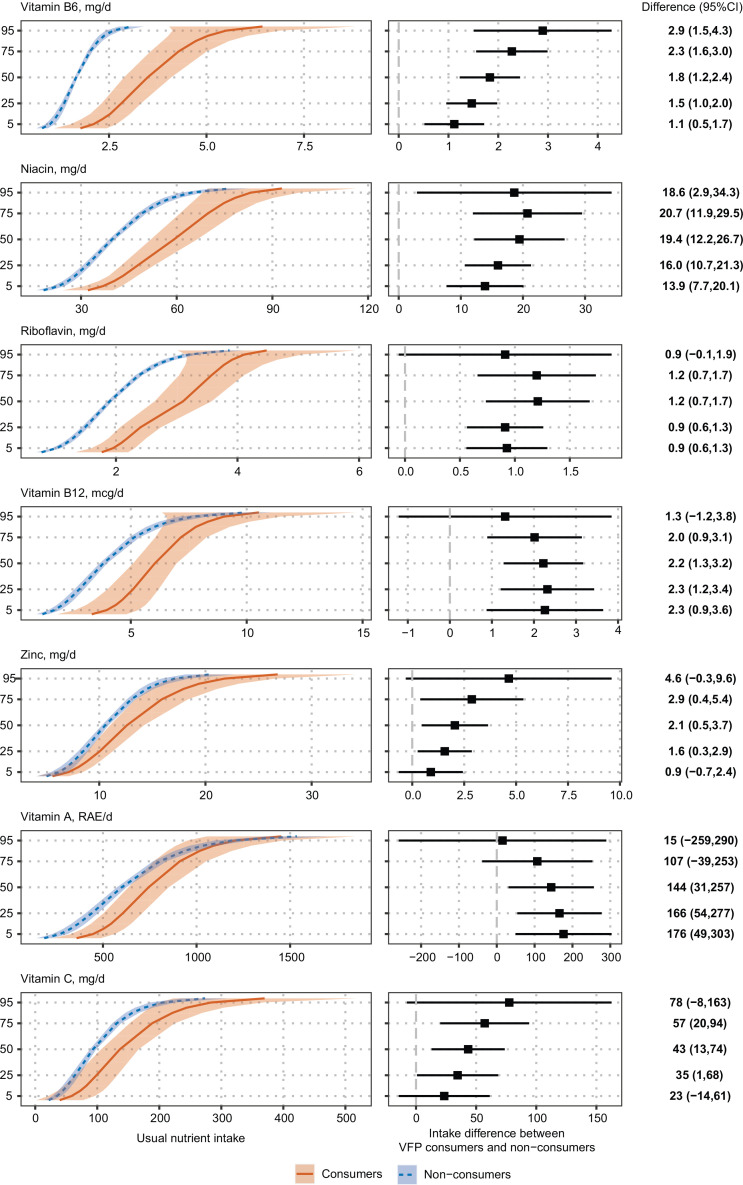
Distributions of usual nutrient intakes for voluntarily fortified products consumers and non-consumers. All data are weighted to be representative of the Canadian population. Shaded bands are 95% confidence intervals (500 bootstrap replicate weights). Plots to the right of these distributions display the difference in the usual intakes of consumers and non-consumers at each centile of the distribution, with 95% confidence intervals. Data above and below the 95th and 5th percentile, respectively, were truncated for clarity since these estimates have very high coefficients of variation.

Figure 3 presents the estimated distributions of usual intake for consumers and non-consumers for each of the micronutrients examined. Supplementary Table 2 presents the corresponding within- and between-person variance estimates and variance ratios for these groups. Despite the wider confidence intervals around the distributions for VFP consumers, the entire distributions of usual intake for niacin and vitamin B6 are significantly higher for VFP consumers than non-consumers, and the 95% confidence intervals only overlap at the 95th centile of the distributions for riboflavin and vitamin B12 and 5th centile of the distribution for zinc. Slightly more overlap is apparent in the 95% confidence intervals of the distributions for vitamins A and C, but the tendency is toward higher usual intakes among consumers. The median usual intakes of VFP consumers were 24–111% higher than the median usual intakes of non-consumers, with the greatest difference observed for vitamin B6 (Supplementary Table 3).

VFP consumers had zero prevalence of inadequate intakes for the four B vitamins assessed (Table 4). Non-consumers also had zero risk of inadequacy for niacin, and although they had significantly higher prevalence of inadequacy for riboflavin and vitamins B6 and B12, their risk was low, with prevalence estimates ranging from 3.3% for riboflavin (95% CI: 1.8–4.9) to 9.1% for vitamin B12 (95% CI: 5.3–13.0). VFP consumers also had significantly lower prevalence of inadequacy than non-consumers for vitamins A and C, but not for zinc. It should be noted, however, that there is a high level of uncertainty associated with the prevalence estimates among consumers for these three nutrients (as indicated by coefficients of variation above 33.3%).

**Table 4:** Prevalence of nutrient inadequacy in 14–50-year-old Canadians, by voluntarily fortified product consumption

Nutrient	Prevalence (95%CI) of nutrient inadequacy		*P* ^b^
	Non-consumers (*n* = 8,135)^a^	Consumers (*n* = 307)^a^	
Vitamin A	43.6 (39.3, 47.9)	21.9 (4.4, 39.3)^c^	0.019
Vitamin C	27.0 (21.3, 32.7)	10.0 (0.0, 24.6)^c^	0.023
Niacin	0 (0, 0)	0 (0, 0)	
Riboflavin	3.3 (1.8, 4.9)	0.0 (0.0, 0.1)	<0.001
Vitamin B6	9.0 (4.2, 13.8)	0.0 (0.0, 1.3)	<0.001
Vitamin B12	9.1 (5.3, 13.0)	0.0 (0.0, 1.3)	<0.001
Zinc	14.7 (6.9, 22.5)	10.0 (1.3, 18.8)^c^	0.474

a Sample sizes indicate the number of respondents (no sampling weights applied). All proportions are weighted to be representative of the Canadian population.

b *P*-value indicates the compatibility of observed difference in the prevalence of nutrient inadequacy between consumers and non-consumers with the (null) hypothesis that the difference is zero.

c Estimate with high uncertainty as per Statistics Canada’s standard (coefficient of variation > 33.3%).

When nutrients from supplements were included in the estimated distributions of usual intake for supplement users (i.e. 29.7% of VFP consumers and 38.3% of non-consumers), VFP consumers who took supplements had higher median usual intakes than non-consumers who took supplements, but the standard errors around these estimates were large (Supplemental Table 4). Irrespective of VFP consumption, supplement users had <5.0% prevalence of inadequacy for the nutrients examined (Supplemental Table 5). However, the 90th centile of usual vitamin A intakes for both VFP consumers and non-consumers who took supplements exceeded the UL of 3,000 µg RAE (Supplemental Table 2). The 95th centile of usual zinc intakes for VFP consumers also approximated the UL (40 mg), and it exceeded the UL among non-consumers who took supplements.

## Discussion

In the context of expanding voluntary fortification in Canada, our results suggest that VFPs contribute substantially to consumers’ micronutrient intakes, disproportionately positioning them in the upper quartile of population intakes for five of the seven micronutrients examined and resulting in markedly elevated distributions of usual nutrient intake for consumers compared to the non-consumer population. Nutrient loads were further elevated by intakes from supplements among almost one-third of consumers.

One defining feature of voluntary fortification in Canada is that it represents a commercial choice, not a public health measure. VFP contributed substantially to consumers’ intakes of commonly added nutrients, but there was little indication that this afforded valuable protection from risks of nutrient inadequacy. Consumers and non-consumers differed most with respect to their usual intakes of the B vitamins assessed, but even non-consumers had a negligible probability of inadequate intakes of these vitamins. While consumers had significantly lower prevalence of inadequate vitamins A and C intakes than non-consumers, the extent to which these differences can be attributed to VFP consumption is unclear from our analyses. Many VFP consumers obtained little or no vitamin A or C from the VFP products they consumed, a finding consistent with a recent Canadian study showing that very few energy drinks contain any vitamin A or C, and the levels of addition to ‘supplemented foods’ are highly variable (10). The VFP consumers in this study tended to be more affluent and more physically active than non-consumers, and there may have been differences in their dietary patterns beyond VFP consumption. More research is needed to determine the contribution of VFP consumption to the observed differences in the prevalence of inadequate vitamins A and C intakes.

Our limited indications that VFP consumption conferred important protection from micronutrient inadequacies differ from the inferences drawn from analyses of the contributions of voluntary fortification to usual intakes in the United States and Ireland (14–17). However, it is important to recognize that population-based studies suggesting benefits of voluntary fortification for nutrient adequacy have occurred in settings where voluntary fortification encompasses nutrient additions for public health reasons. Exposure to VFP is consequently much more pervasive in these populations. Our findings pertain only to the effects of fortification in Canada that has occurred for marketing purposes, with no public health rationale; we have focused on the consumption of a very limited selection of products. The nutrients permitted for addition, the permissible levels of addition, and the food vehicles chosen for voluntary fortification differ markedly from the much more tightly controlled mandatory fortification programs implemented to address public health concerns in Canada. In this regulatory context, the benefits of voluntary fortification are not obvious.

Our analyses revealed little evidence of safety concerns based on conventional reference values. The upper tails of the estimated distributions of usual vitamin A and zinc intake exceeded ULs for VFP consumers who also took supplements, but their high intakes appear to be more a function of nutrient loads from supplements than VFP consumption, given that the distributions of non-consumers who took supplements also abutted or exceeded the ULs for these nutrients. While this analysis would suggest that the VFP consumption currently does not pose any safety risks to the Canadian population, a recent examination of the nutrient content of voluntarily fortified beverages indicated that most manufacturers were adding less than half of the nutrient amounts permitted under current regulations (10). As new products continue to be launched and existing products reformulated in this highly competitive industry (12), there is the potential for increased micronutrient fortification.

In interpreting our results, it is also important to recognize the limitations of the ULs. Although based on the best available evidence at the time, these values are not an assurance that chronic intakes at any lower level are without risk (36). Yet, they are the basis for regulatory decisions about maximum levels of addition. For nutrients for which no UL has been established (e.g. pantothenic acid), voluntary fortification is permitted in Canada with no limits (6, 8). The long-term health implications of chronic exposure to multiple micronutrients at levels far above those attained from diets without VFP are essentially unknown.

## Limitations

Our estimated prevalence of the VFP consumption in the Canadian population based on a single 24-h recall must understate total consumption in the population insofar as we have limited capture of occasional consumption. It is impossible to gauge the extent of this bias from the data available to us. Sales and consumption levels for energy drinks, ‘vitamin waters’, and other novel beverages have risen in North America (37, 38), and there is evidence of a steady increase in the sale of energy drinks since these products were first approved in Canada (12, 13). The use of these products may be sporadic or episodic in nature and, therefore, prone to underestimation with single 24-h recalls, but these products may also be subject to reporting errors. Very few VFPs are in the Canadian Nutrient File 2015, and given that the computer-assisted recall interview was directly linked to this database, probing during the 24-h recalls may have been insufficient to enable the identification of the full range of products currently available. The discrepancy between market sales data for sugar-sweetened beverages in Canada and their reported consumption on CCHS 2015 (13), when considered in tandem with the documented problem of energy underreporting on this survey (24), also raises the possibility that voluntarily fortified beverage intakes were underreported.

The small number of survey participants reporting any consumption of VFP in this survey precluded analyses to isolate the influence of nutrients obtained from VFP on consumers’ probability of inadequate nutrient intakes. The small number of VFP consumers identified also precluded analysis of the effects of individual products or product groups on total nutrient exposure. Given differences in current Canadian regulations governing the selection and maximum levels of nutrients permissible for addition to energy drinks and other products (6, 8), and differences in industry norms regarding the selection of nutrients for different product lines (10, 11) and the promotion of these products, habitual intake of different VFPs could be expected to impact usual nutrient intakes differently.

Our estimation of separate usual intake distributions for consumers and non-consumers was necessitated by the very low prevalence of VFP consumption reported by survey participants. With such a high proportion of zero intakes, it was not feasible to analyze VFP as an episodically consumed food (39, 40). Our bifurcation of the sample into consumers and non-consumers based on their intake behaviors on a single day undoubtedly resulted in misclassification error with respect to habitual consumption, which, in turn, may have biased our estimated distributions of usual intake. The much higher proportion of VFP consumers reporting VFP intakes on both 24-h recalls suggests that the habitual VFP consumption is concentrated in a discrete population subgroup. However, food frequency data are needed to verify this and better characterize the consumer group.

The limited number of VFP included in the Canadian Nutrient File meant that the true variation in nutrient additions among the products sold in Canada was suppressed. Both the selection and amounts of micronutrient additions have been found to vary substantially across different varieties of energy drinks and fortified beverages (10, 11), and new products continue to be launched in this highly competitive market (12). A comparison of the Canadian Nutrient File entries for energy drinks to the nutrient composition of leading brands in Canada (10) indicates substantial underestimation of some nutrient exposures. This observation lends support to the calls of other authors for brand information to be included in both consumption and composition data (2).

## Implications for monitoring and regulation

Given the low reported consumption of VFP, it is important to append food frequency questions about VFP intakes to future population intake surveys to facilitate fuller analysis of their effects on usual intakes in Canada. Our results also highlight the need for more detailed, brand-specific data on the consumption and micronutrient composition of these products. In the absence of such data, dietary assessments are biased toward underestimation, impeding identification of risks of excessive intakes.

It is a common practice for regulatory agencies to base determinations of maximum allowable nutrient additions for voluntarily fortified foods and beverages on estimates of the 95th percentile of the distribution of usual intakes in the entire population (3). Once regulations are implemented and VFP begin to penetrate the marketplace, our results suggest that only a fraction of the population may consume them, yet their nutrient intakes are significantly elevated by that practice. This observation highlights the importance of considering the nutrient exposure levels of consumers and non-consumers within the population separately when setting regulations for voluntary fortification and monitoring their impact relative to risks of excessive intakes.
